# Impact of comfort theory–guided nursing care on anxiety reduction and recovery outcomes in pediatric strabismus surgery patients

**DOI:** 10.3389/fped.2026.1766851

**Published:** 2026-04-27

**Authors:** Tingting Liu, Qing Liao

**Affiliations:** Central Operating Room, Nanjing Tongren Hospital, School of Medicine, Southeast University, Nanjing, China

**Keywords:** anxiety, children, comfort theory–guided nursing care, outcomes, recovery, strabismus

## Abstract

**Objective:**

This study aimed to evaluate the effectiveness of Comfort Theory–guided nursing care in reducing perioperative anxiety and improving recovery outcomes among children undergoing strabismus surgery. The hypothesis was that a structured comfort-based nursing approach addressing physical, psychospiritual, sociocultural, and environmental needs would yield superior emotional and functional outcomes compared with conventional care.

**Methods:**

A prospective study was conducted involving 184 pediatric patients scheduled for elective strabismus correction. Participants were randomly assigned to receive either routine nursing (*n* = 92) or Comfort Theory–guided nursing interventions (*n* = 92). The comfort-based model included individualized psychological preparation, environmental optimization, parental involvement, and continuous emotional support. Anxiety was measured using the Modified Yale Preoperative Anxiety Scale (m-YPAS), pain using the Face–Legs–Activity–Cry–Consolability (FLACC) scale, and behavioral recovery using the Post-Hospitalization Behavior Questionnaire (PHBQ).

**Results:**

Compared with routine care, the Comfort Theory group showed markedly lower anxiety both before induction (44.1 ± 8.9 *vs.* 61.5 ± 9.7, *p* < 0.001) and on postoperative day 1 (40.6 ± 7.5 *vs.* 53.4 ± 8.2, *p* < 0.001). Physiological stress responses were also blunted, with lower heart rate (100.2 ± 8.8 *vs.* 108.5 ± 9.2 bpm, *p* < 0.001) and mean arterial pressure (83.1 ± 6.7 *vs.* 88.4 ± 7.1 mmHg, *p* < 0.001) before induction. Postoperative pain scores were significantly reduced across all time points (*p* < 0.001), and behavioral recovery improved, with lower PHBQ scores (7.4 ± 3.1 *vs.* 10.2 ± 3.7, *p* < 0.001). Children in the intervention group regained consciousness earlier (19.6 ± 3.8 *vs.* 22.4 ± 4.1 min, *p* < 0.001), resumed oral intake faster (4.9 ± 1.0 *vs.* 5.8 ± 1.2 h, *p* < 0.001), and had shorter hospital stays (3.4 ± 0.7 *vs.* 3.9 ± 0.8 days, *p* < 0.001).

**Conclusion:**

Comfort Theory–guided nursing care may help alleviate perioperative anxiety, stabilize physiological stress responses, reduce postoperative pain, and support behavioral and functional recovery in pediatric strabismus surgery. As a holistic nursing approach, it may offer a practical framework for improving perioperative care; however, the findings should be interpreted in light of the single-center design, lack of full blinding, and multimodal nature of the intervention.

## Introduction

Strabismus, commonly referred to as ocular misalignment, is one of the most prevalent visual disorders in childhood, affecting approximately 2%–4% of the pediatric population worldwide (1). Surgical correction remains the primary treatment option for restoring binocular function, ocular alignment, and psychosocial confidence in affected children (2, 3). However, despite advances in surgical techniques and anesthesia, perioperative anxiety and postoperative distress remain major challenges in pediatric ophthalmic surgery (4). Preoperative anxiety can provoke physiological stress responses such as tachycardia, elevated cortisol levels, and increased anesthetic requirements. It has also been associated with prolonged recovery, greater postoperative pain, and maladaptive behavioral changes (5, 6). The heightened sensitivity of children to environmental stimuli, coupled with their limited coping capacity, makes anxiety management a critical determinant of perioperative safety and recovery quality (7). Therefore, nursing interventions that holistically address psychological comfort and emotional well-being, rather than focusing solely on physiological parameters, are increasingly recognized as integral to optimizing pediatric surgical outcomes (8).

The concept of comfort in nursing care was formally introduced by Katharine Kolcaba in the 2000s as a middle-range theory describing comfort as an immediate, holistic experience of being strengthened through relief, ease, and transcendence in physical, psychospiritual, sociocultural, and environmental contexts (9). Comfort Theory provides a structured framework for assessing and fulfilling patients' multifaceted needs, emphasizing that comfort is not merely the absence of distress but the presence of a positive state that facilitates healing and adaptation (10). In pediatric perioperative care, the application of Comfort Theory allows nurses to design interventions that integrate physical soothing (e.g., pain and temperature management), psychological reassurance (e.g., anxiety relief, emotional security), and social engagement (e.g., parental presence, communication support) (11). By meeting these diverse needs, nursing care grounded in Comfort Theory may create an emotionally safe environment that attenuates stress responses, strengthens coping mechanisms, and promotes faster recovery (6). Previous research in adult surgical or chronic care populations has demonstrated that comfort-oriented nursing significantly reduces anxiety, improves satisfaction, and enhances adherence to treatment regimens (9). Yet, despite the conceptual appeal and proven benefits in adult settings, evidence on the implementation and efficacy of Comfort Theory–based interventions in pediatric surgical care remains limited and fragmented.

In the specific context of ophthalmic surgery, pediatric patients undergoing strabismus correction are uniquely vulnerable to anxiety and postoperative distress for several reasons. First, the procedure involves manipulation of the ocular region, which is a highly sensitive and visually salient organ and may evoke disproportionate fear even in otherwise calm children. Second, perioperative visual obscuration caused by bandages or temporary diplopia can induce confusion, irritability, and noncooperation during recovery. Third, communication barriers between children and healthcare providers often hinder accurate expression of fear or pain, leading to unrecognized anxiety and inadequate emotional support. Studies have shown that over 70% of children experience moderate-to-severe anxiety before ophthalmic surgery, and nearly half exhibit behavioral regression or sleep disturbances postoperatively (12, 13). Conventional nursing care typically focuses on preoperative explanations, routine monitoring of vital signs, and medication administration and may not adequately address these complex psychosocial dimensions (5). In contrast, Comfort Theory–guided nursing interventions aim to integrate emotional reassurance, sensory comfort, and parental involvement as part of routine perioperative management, thereby potentially transforming the child's experience from one of fear and helplessness to trust and cooperation (14). For example, techniques such as individualized psychological preparation, child-friendly environmental modification, and structured parental participation during induction have shown promise in mitigating preoperative distress (8). However, such interventions are rarely standardized or theoretically anchored in current pediatric ophthalmic practice.

Recent pediatric nursing studies have increasingly highlighted the role of theory-based care models in improving emotional regulation and recovery outcomes. Frameworks such as family-centered care, humanistic nursing, and Comfort Theory have been shown to enhance child–nurse communication, parental satisfaction, and postoperative adaptation (15). Comfort Theory, in particular, offers the advantage of operational clarity through its three forms of comfort (relief, ease, transcendence) and four contexts (physical, psychospiritual, sociocultural, environmental), enabling systematic assessment and measurable intervention outcomes. Empirical evidence from pediatric surgery units applying this model has reported reductions in anxiety scores, pain intensity, and length of hospital stay (11). Nevertheless, most prior studies were limited by small sample sizes, single-dimension outcome measures, or lack of control groups, leaving uncertainty about the causal impact of Comfort Theory–guided care on objective recovery indicators (16). Moreover, its applicability to highly anxiety-provoking procedures such as strabismus surgery has not been thoroughly evaluated. Given that emotional state and cooperation significantly influence surgical success and visual rehabilitation in children, integrating Comfort Theory into the perioperative care of strabismus surgery patients may yield substantial clinical and psychological benefits (17).

This study, therefore, sought to investigate the impact of Comfort Theory–guided nursing interventions on anxiety reduction and recovery outcomes in pediatric patients undergoing strabismus surgery. We hypothesized that a structured comfort-based nursing approach would result in significantly lower perioperative anxiety, improved cooperation during induction and recovery, reduced postoperative pain, and shorter recovery duration compared with conventional routine care. By quantitatively assessing both subjective (e.g., anxiety scales) and objective (e.g., recovery time, complication rates) indicators, this research aimed to generate evidence supporting the integration of Comfort Theory into standard pediatric ophthalmic nursing protocols. The novelty of this study lies in the comprehensive application of a holistic nursing model in a pediatric surgical setting, an area where evidence remains limited, as well as in its focus on linking emotional comfort with measurable clinical outcomes. Furthermore, the study contributes to the theoretical advancement of nursing science by empirically testing Comfort Theory in a vulnerable population with distinctive emotional and physiological stress responses. Findings from this investigation may provide a practical framework for nursing educators and clinical managers to design evidence-based comfort care programs, ultimately enhancing both patient experiences and postoperative recovery trajectories in pediatric ophthalmology.

## Methods

### Ethical approval and compliance

Written informed consent was obtained from all participants. This research was performed in accordance with the Declaration of Helsinki and was approved by Nanjing Tongren Hospital. Written informed consent was obtained from the parents or legal guardians of all participants prior to enrollment. Children were informed of the procedure in age-appropriate language to ensure comprehension and voluntary cooperation. No deviation from the approved protocol was made during the study period.

### Study design and participants

A prospective, parallel-group, controlled clinical study was conducted from January 2022 to June 2024 in the Department of Ophthalmology, Nanjing Tongren Hospital. A total of 184 pediatric patients scheduled for elective strabismus correction surgery under general anesthesia were consecutively recruited. Participants were randomly assigned in a 1:1 ratio to either the control group (*n* = 92), receiving conventional routine nursing care, or the intervention group (*n* = 92), receiving Comfort Theory–based nursing care.

Randomization was performed using a computer-generated random sequence prepared by an independent statistician who was not involved in patient recruitment, clinical care, or outcome assessment. Allocation concealment was ensured using sequentially numbered, opaque, sealed envelopes, which were opened only after participant enrollment.

**Figure 1:**
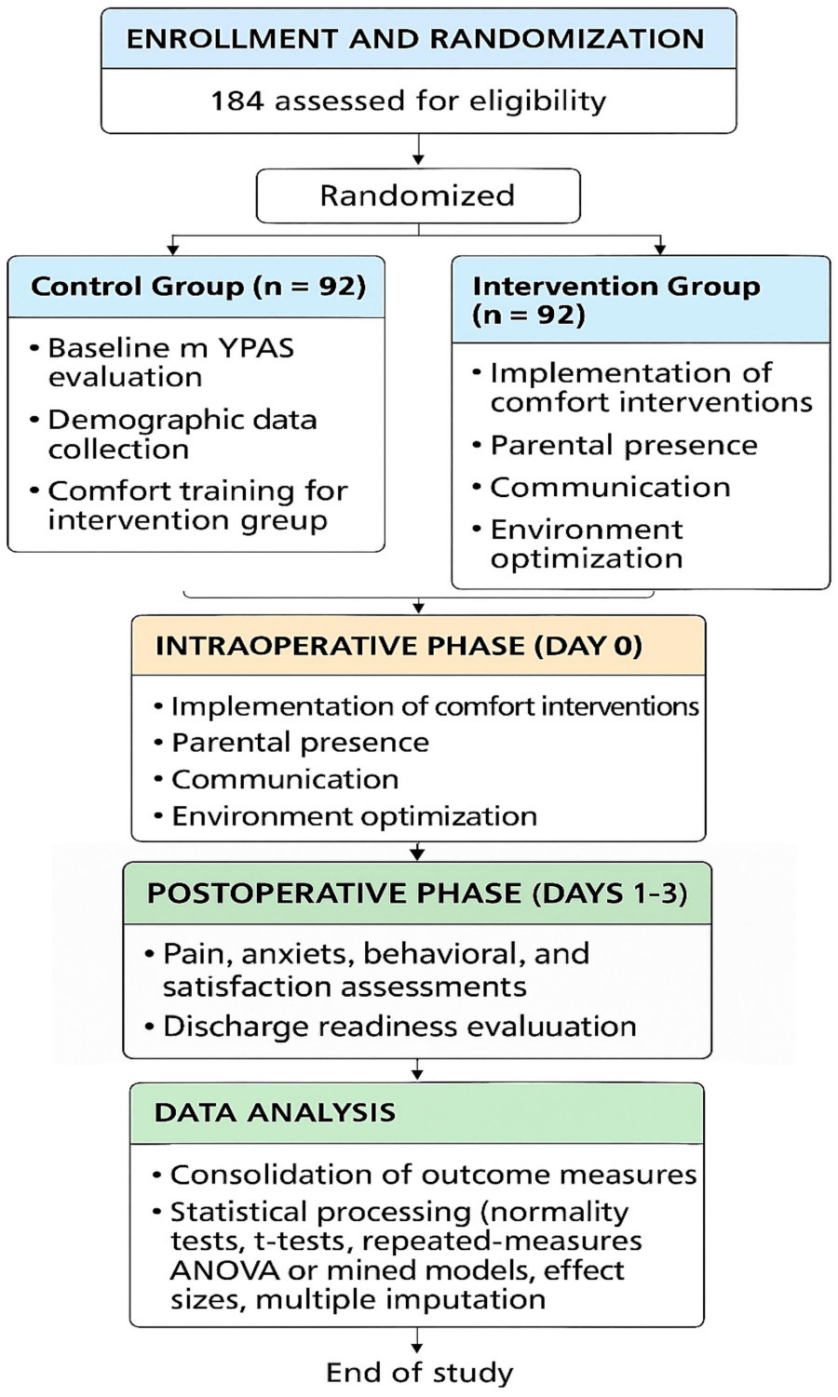
Schematic representation of the study design and participant flow. Overview of the study design and participant allocation. A total of 184 pediatric patients were randomized 1:1 to receive either routine nursing care or Comfort Theory–based nursing care. The schematic outlines the preoperative, intraoperative, and postoperative phases with key evaluation points at baseline, postoperative day 1, and day 3.

The study followed a single-blind design in which outcome assessors responsible for administering the behavioral scales were unaware of group assignments. Due to the nature of the nursing intervention, blinding of patients, parents, and nursing staff was not feasible. This introduces a potential risk of performance bias and expectation bias, particularly given that behavioral and observer-dependent outcomes were assessed. To mitigate this limitation, outcome evaluations were conducted using standardized and validated instruments by trained assessors who were blinded to group allocation and not involved in intervention delivery. Figure 1 illustrates the overall study schematic, including patient recruitment, group allocation, perioperative intervention timeline, and outcome evaluation points (preoperative baseline, postoperative day 1, and postoperative day 3). Inclusion criteria: (1) Children aged 4–12 years diagnosed with comitant or partially accommodative strabismus confirmed by an ophthalmologist; (2) Scheduled for primary unilateral or bilateral extraocular muscle surgery; (3) Classified as American Society of Anesthesiologists (ASA) physical status I–II; (4) Normal cognitive development and ability to understand instructions; (5) Written informed consent from parent/guardian. Exclusion criteria: (1) Previous ocular surgery or trauma; (2) Neurological or psychiatric disorders (e.g., epilepsy, autism, or developmental delay); (3) Severe systemic disease (cardiac, hepatic, or renal insufficiency); (4) Current use of psychotropic medications; (5) Incomplete perioperative data or refusal to participate in follow-up.

### Sample size calculation

Sample size was estimated based on preoperative anxiety score reduction as the primary endpoint. Assuming an effect size (d) of 0.6, a power (1—*β*) of 0.80, and *α* = 0.05, a minimum of 80 participants per group was required. Considering potential attrition of 15%, 92 subjects were recruited per group, yielding a total of 184 cases for analysis. Statistical calculation was performed using G*Power 3.1.

### Preoperative assessment and baseline data collection

Baseline demographic and clinical information, including age, sex, type of strabismus, surgical plan, and anesthesia duration, was collected from electronic medical records. Preoperative anxiety was measured the evening before surgery using the Modified Yale Preoperative Anxiety Scale (m-YPAS) administered by a trained pediatric nurse blinded to group assignment (18). The scale assesses five domains (activity, vocalization, emotional expressivity, state of arousal, and use of parents) on a 4-point Likert system; higher scores indicate greater anxiety. Baseline vital signs (heart rate, systolic and diastolic blood pressure, and oxygen saturation) were recorded using a calibrated patient monitor.

### Perioperative nursing interventions

Participants in the control group received conventional perioperative nursing care according to the hospital's standardized clinical pathway for pediatric ophthalmic surgery. This routine care mainly included preoperative health education provided to parents, environmental maintenance of the ward (temperature 24 ± 1°C, quiet and clean surroundings), routine monitoring of vital signs, basic emotional reassurance, and standard postoperative pain and antiemetic management according to physician orders. No structured psychosocial or environmental comfort measures were incorporated into the routine nursing model.

In contrast, patients in the intervention group received comprehensive nursing care guided by Kolcaba's Comfort Theory, which emphasizes the attainment of comfort through the dimensions of physical, psychospiritual, sociocultural, and environmental needs (19). Before the initiation of the study, all nurses in the intervention team underwent a two-week training program on Comfort Theory principles, communication techniques with children, and standardized comfort intervention procedures to ensure consistency and fidelity of implementation. The intervention protocol specified the timing, duration, and key components of each comfort measure, ensuring that psychological preparation, environmental adjustments, parental participation, and emotional-support strategies were implemented consistently across participants. The Comfort Theory–based care model was delivered continuously throughout the perioperative period, including preoperative preparation, intraoperative support, and postoperative recovery phases.

During the preoperative stage, nurses conducted individualized comfort assessments to identify each child's anxiety triggers, preferred soothing methods, and coping style. Physical comfort was enhanced by optimizing the ward environment, including adjustment of light intensity, noise levels, and temperature, and by providing warm blankets and gentle tactile reassurance during the preoperative waiting period. To relieve psychospiritual tension, nurses engaged children in age-appropriate psychological preparation, such as using illustrated story cards and cartoon animations to explain the surgical process, thereby converting unfamiliar medical procedures into understandable narratives. Coping rehearsal was encouraged through guided breathing and relaxation training, while therapeutic communication strategies, including eye-level contact and positive reinforcement, were applied to build trust and reduce fear. Parents were encouraged to participate in the education sessions, which provided instructions on how to maintain calm behavior, use verbal reassurance, and avoid transmitting their own anxiety to the child.

During the intraoperative period, comfort-based nursing measures focused on emotional stability and environmental control. Nurses accompanied children to the operating area, maintaining physical proximity and verbal reassurance until anesthesia induction. Parental presence was permitted during anesthesia induction to provide emotional continuity and mitigate separation anxiety. The ambient light and noise levels in the operating waiting area were controlled to create a calm environment. Nurses closely observed the children's nonverbal cues, such as muscle tension, facial expressions, or crying, and provided comfort through gentle verbal communication or distraction techniques (e.g., holding toys or storytelling). All comfort interventions were documented in the nursing log immediately after completion.

In the postoperative phase, the comfort care approach continued to address both physical and emotional recovery needs. Nurses applied a standardized postoperative analgesia protocol that was identical for both groups, in conjunction with continuous pain assessment using the Face, Legs, Activity, Cry, Consolability (FLACC) scale at 1, 6, 12, and 24 h post-surgery. Analgesic medication was administered according to hospital guidelines when the FLACC score exceeded 3, ensuring consistent pain management across participants. Analgesic supplementation was provided when FLACC scores exceeded 3. To reduce psychospiritual distress, nurses reassured children immediately after awakening from anesthesia, explained the purpose of eye bandages in simple language, and gently corrected misinterpretations or fears. Parents were allowed to stay at the bedside during recovery to enhance security and minimize crying episodes. Play therapy, including the use of small toys, drawing materials, and music, was used to divert attention from discomfort and improve mood. The postoperative environment was maintained quiet and softly illuminated, and visual or auditory stimulation was minimized during early recovery to prevent agitation. Nurses continued to provide emotional comfort through verbal praise, physical touch, and positive reinforcement whenever the child demonstrated cooperation with eye protection or medication administration.

All interventions were delivered according to pre-defined comfort dimensions and were evaluated daily for adherence. Supervising nurse educators audited comfort implementation logs every two weeks to ensure procedural consistency. The comfort care process was documented in the nursing records with detailed notes on specific interventions, the child's responses, and the duration of each session. These records were subsequently reviewed by the research coordinator for completeness and fidelity verification. Through this integrated and theory-driven nursing approach, the intervention aimed to achieve holistic comfort by simultaneously alleviating physiological discomfort, reducing anxiety, enhancing family participation, and optimizing the perioperative environment, thereby promoting a smoother psychological and physical recovery trajectory after pediatric strabismus surgery.

### Surgical and anesthetic procedure

All surgeries were performed by the same ophthalmic surgical team to minimize procedural variability. General anesthesia was induced using intravenous propofol and maintained with sevoflurane inhalation. No routine sedative premedication, including midazolam, was administered before anesthesia induction to avoid confounding effects on perioperative anxiety assessment. Standard intraoperative monitoring included electrocardiography, non-invasive blood pressure, pulse oximetry, and end-tidal CO₂. Surgical duration, the number of extraocular muscles operated, and intraoperative events were recorded to evaluate procedural comparability between groups. Postoperative recovery was supervised in the pediatric ophthalmic ward under identical medical protocols for both groups, differing only in nursing approach.

### Outcome measures

The outcome evaluation framework was designed to comprehensively assess both psychological and physiological effects of the nursing intervention. The primary outcome was the change in preoperative anxiety levels, while secondary outcomes captured pain intensity, behavioral adaptation, physiological stress responses, parental satisfaction, and functional recovery indicators, including awakening time, time to oral intake, and length of hospital stay. All outcome measurements were performed by two trained assessors who were blinded to group allocation to minimize observer bias.

Perioperative anxiety was measured using the m-YPAS at three time points: the evening before surgery (baseline), immediately before anesthesia induction, and on postoperative day one. The m-YPAS evaluates observable anxiety behaviors in children across five domains: activity, vocalization, emotional expressivity, state of arousal, and use of parents. The total score ranges from 23 to 100, with higher scores indicating greater anxiety. A mean reduction in m-YPAS from baseline to pre-induction was considered the principal indicator of intervention efficacy.

Physiological stress responses were recorded synchronously with anxiety assessments. Heart rate and mean arterial pressure were measured using an automated vital-sign monitor at baseline, immediately before induction, five minutes after induction, and upon arrival in the recovery room. These indices were used as objective correlates of stress activation.

Postoperative pain was evaluated using the Face, Legs, Activity, Cry, Consolability (FLACC) behavioral pain scale at 1, 6, 12, and 24 h after surgery. The FLACC scale, validated for nonverbal children, rates each behavioral dimension from 0 to 2, with a total score of 0–10; values above 3 prompted supplemental analgesia per hospital protocol.

Behavioral adaptation following hospitalization was assessed on postoperative day three using the Post-Hospitalization Behavior Questionnaire (PHBQ), which captures negative behavioral changes across domains such as anxiety, regression, and sleep disturbance. Higher PHBQ scores indicate poorer postoperative adaptation.

To reflect functional and recovery outcomes, the study additionally evaluated time to awakening (interval from cessation of anesthetics to full consciousness) and time to first oral intake. These indices were recorded directly from anesthetic and nursing logs. Total length of hospital stay (LOS) from admission to discharge was documented as an integrative recovery indicator.

Finally, parental satisfaction was surveyed using a five-item Likert questionnaire developed by the hospital's pediatric nursing quality team. The questionnaire assessed satisfaction with nursing communication, comfort interventions, perceived empathy, recovery support, and overall care experience. Scores ranged from 1 (very dissatisfied) to 5 (very satisfied). All questionnaires were administered face-to-face by independent staff who were not involved in patient care. As this questionnaire was developed for internal quality assessment, it has not undergone formal external validation and the corresponding findings should therefore be interpreted as supportive rather than definitive.

### Quality control and training

Before study commencement, all participating nurses underwent a two-week standardized training on Comfort Theory principles, psychological communication, and play therapy techniques. Training completion was verified through a competency test and supervised bedside evaluation. To ensure reliability, inter-rater consistency for m-YPAS and FLACC scoring was tested in 20 pilot cases (intraclass correlation coefficient = 0.91). Weekly review meetings were held to discuss adherence issues and ensure protocol consistency.

### Safety and adverse event monitoring

All adverse events, including postoperative nausea, vomiting, emergence agitation, and wound infection, were recorded and managed according to hospital guidelines. Serious events were reported to the ethics committee within 24 h. No protocol-related harm occurred during the study.

### Data analysis

All collected data were double-entered and verified by two independent research assistants to ensure accuracy and completeness. Statistical analyses were performed using SPSS version 27.0. Continuous variables were examined for normality using the Shapiro–Wilk test. Normally distributed data were expressed as mean ± standard deviation (SD), while non-normally distributed variables were described as median (interquartile range). Categorical variables were summarized as counts and percentages. Comparisons between the intervention and control groups at baseline were conducted using independent-sample *t*-tests for continuous variables and *χ*2 tests or Fisher's exact tests for categorical variables. Changes in repeated measurements over time, including anxiety (m-YPAS), heart rate, and mean arterial pressure, were analyzed using repeated-measures analysis of variance (ANOVA), with Greenhouse–Geisser correction applied when necessary. When significant interactions were observed, *post-hoc* Bonferroni tests were applied to identify pairwise differences. To reduce the risk of inflated type I error in repeated pairwise comparisons, Bonferroni correction was applied for *post-hoc* analyses of time-dependent outcomes. Other secondary outcome analyses were considered supportive and were interpreted cautiously in light of multiple testing. Effect sizes were calculated to assess the magnitude of clinical impact: Cohen's d was reported for continuous outcomes and Cramer's V for categorical comparisons. Pain trajectories derived from FLACC scores were evaluated using linear mixed-effects modeling to account for within-subject correlations and missing repeated data. Behavioral recovery and satisfaction outcomes were compared using the Mann–Whitney *U*-test when distributional assumptions were violated. Missing data less than 5% of total observations were imputed using multiple-imputation procedures (five iterations) under the assumption of missing at random. Sensitivity analyses confirmed that the results remained stable under complete-case analysis. Statistical significance was set at a two-tailed *p* < 0.05.

## Results

### Participant enrollment and baseline characteristics

A total of 184 pediatric patients undergoing elective strabismus surgery were enrolled between January 2022 and June 2024, with 92 patients assigned to each group. No participant withdrew or was lost to follow-up, yielding a 100% data completion rate. As shown in Table 1, baseline demographic and clinical variables were comparable between groups (all *p* > 0.05). The mean age was 7.1 ± 2.1 years (range, 4–12 years), and 52.2% of participants were male. The distribution of strabismus type (comitant vs. partially accommodative), ASA physical classification, number of extraocular muscles operated, anesthesia duration, and surgical duration showed no statistically significant differences between groups. Baseline anxiety (m-YPAS) and preoperative FLACC pain scores were also similar (*p* = 0.698 and *p* = 0.303, respectively), confirming the homogeneity of both cohorts prior to intervention. These findings ensured that subsequent outcome differences were attributable to the nursing intervention rather than baseline variability.

**Table 1:** Baseline demographic and clinical characteristics of pediatric strabismus surgery patients.

Variable	Intervention group (*n* = 92)	Control group (*n* = 92)	t/*χ*^2^	*p*-value
Age (years, mean ± SD)	7.1 ± 2.0	7.2 ± 2.1	−0.333	0.740
Sex (male/female)	47/45	49/43	0.087	0.768
Type of strabismus (Comitant/Partially accommodative)	66/26	63/29	0.233	0.629
ASA Class (I/II)	58/34	56/36	0.092	0.761
Duration of anesthesia (min)	77.2 ± 9.8	78.5 ± 10.4	−0.829	0.409
Surgery duration (min)	51.7 ± 6.8	52.3 ± 7.1	−0.561	0.576
Baseline m-YPAS	46.2 ± 8.1	45.7 ± 8.3	0.389	0.698
Baseline FLACC	0.6 ± 0.5	0.7 ± 0.6	−1.040	0.303

This table summarizes the demographic and perioperative baseline data of 184 pediatric patients undergoing elective strabismus surgery. No significant differences were observed between the control and intervention groups in age, sex distribution, type of strabismus, ASA classification, or baseline anxiety and pain scores (all *p* > 0.05), confirming group comparability at study entry. ASA, American Society of Anesthesiologists; m-YPAS, Modified Yale Preoperative Anxiety Scale; FLACC, Face–Legs–Activity–Cry–Consolability pain scale.

### Effect of comfort theory–guided nursing on perioperative anxiety

**Figure 2:**
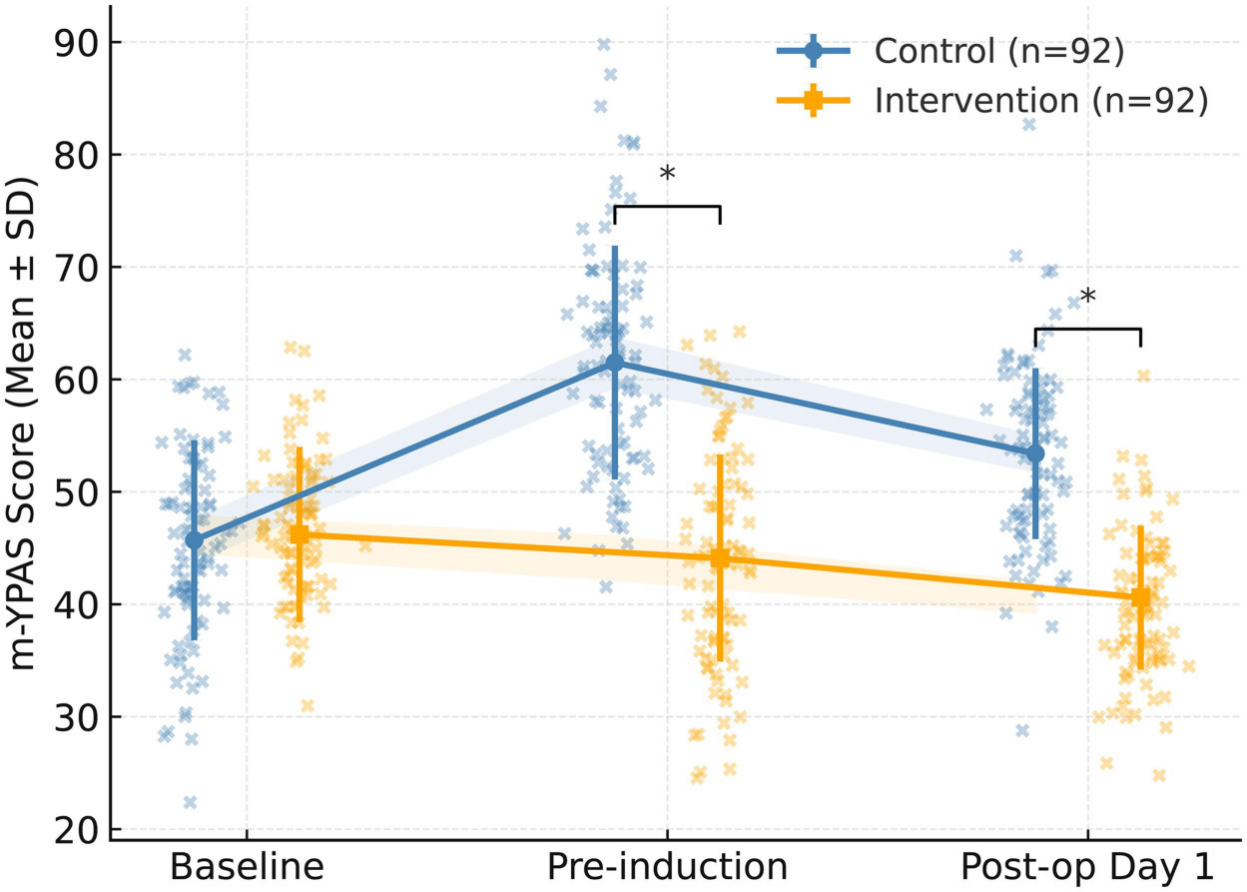
Changes in perioperative anxiety levels between groups assessed by the modified Yale preoperative anxiety scale (m-YPAS). Mean m-YPAS scores were compared between the control and intervention groups at baseline, immediately before anesthesia induction, and on postoperative day 1. The intervention group showed a greater reduction in anxiety over time (group  ×  time interaction *F* = 21.413, *p* < 0.001). Anxiety was significantly lower in the intervention group both before induction and on postoperative day 1. * indicates *p* < 0.05.

Repeated-measures ANOVA revealed a significant group  ×  time interaction in perioperative m-YPAS scores (*F* = 21.413, *p* < 0.001), indicating that anxiety levels evolved differently between groups over time (Figure 2). At baseline, mean m-YPAS scores were nearly identical between the intervention and control groups (46.2 ± 8.1 *vs.* 45.7 ± 8.3; *t* = 0.389, *p* = 0.698). However, immediately before anesthesia induction, the intervention group exhibited markedly lower anxiety (44.1 ± 8.9) compared with controls (61.5 ± 9.7; *t* = −12.049, *p* < 0.001, Cohen's d = 1.80). This trend persisted postoperatively, with m-YPAS scores remaining significantly lower on day 1 after surgery (40.6 ± 7.5 *vs.* 53.4 ± 8.2; *t* = −10.946, *p* < 0.001, Cohen's d = 1.61). *post-hoc* Bonferroni correction confirmed significant differences at both pre-induction and postoperative day 1 (adjusted *p* < 0.001). These results demonstrate that Comfort Theory–guided nursing care substantially attenuated perioperative anxiety and improved emotional stability throughout the surgical period. However, given the behavioral nature of the outcome measures and the absence of full blinding, the magnitude of these effects should be interpreted cautiously. Notably, the observed effect sizes for anxiety reduction (Cohen's *d* > 1.5) are relatively large compared with those typically reported for non-pharmacological interventions. This may be partly explained by the use of observer-based behavioral scales, the continuous and high-intensity nature of the intervention, and contextual influences such as increased caregiver attention and parental involvement. Therefore, the possibility of effect size inflation cannot be excluded.

### Attenuation of physiological stress responses

**Figure 3:**
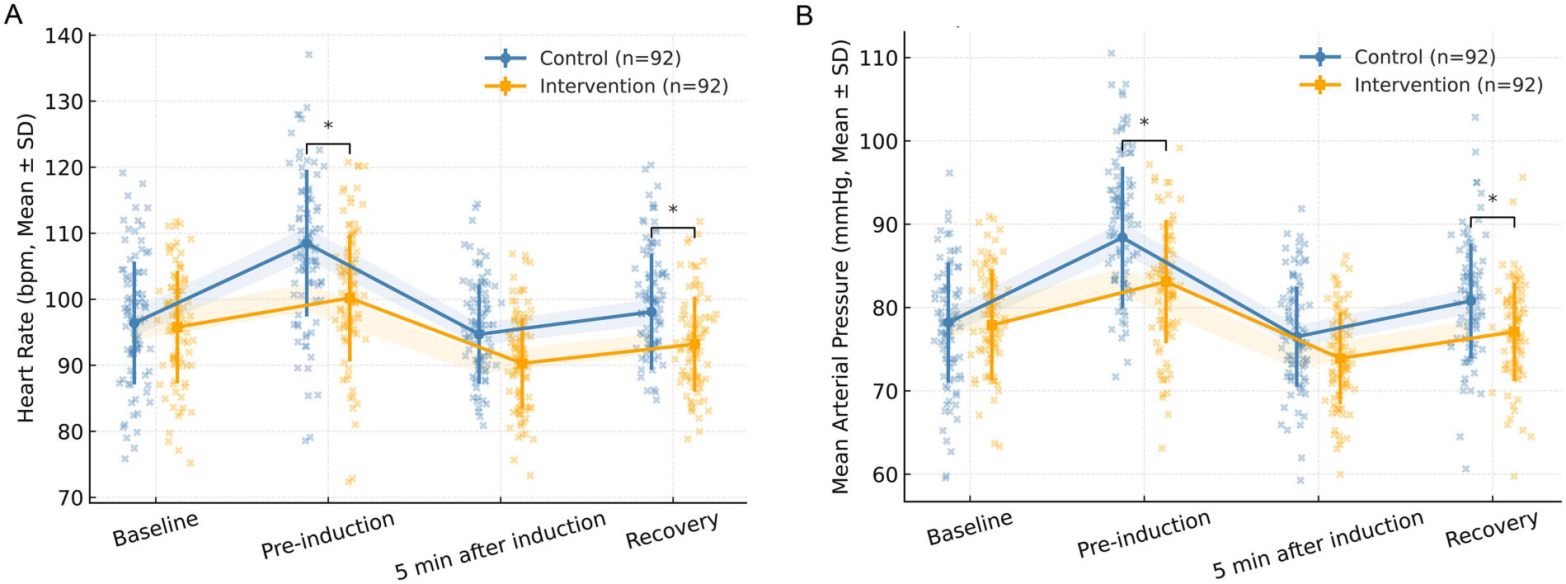
Comparative trends in perioperative physiological stress responses in heart rate (HR) and mean arterial pressure (MAP). Trends in HR and MAP were recorded at baseline, pre-induction, 5 min after induction, and during recovery. The intervention group maintained lower HR and MAP throughout the perioperative period (HR interaction *F* = 8.715, *p* < 0.001; MAP interaction *F* = 6.283, *p* < 0.001). HR and MAP elevations were blunted in the intervention group at pre-induction. * indicates *p* < 0.05.

Physiological indicators mirrored the psychological findings. As displayed in Figure 3, both heart rate (HR) and mean arterial pressure (MAP) rose significantly before induction in both groups, reflecting anticipatory stress, but the magnitude of increase was consistently smaller in the intervention group. Repeated-measures ANOVA demonstrated significant main effects of time (*F* = 36.218, *p* < 0.001) and group (F = 24.879, *p* < 0.001), as well as a group  ×  time interaction (*F* = 8.715, *p* < 0.001) for HR. Similarly, MAP revealed significant group differences (interaction: *F* = 6.283, *p* < 0.001). At pre-induction, mean HR and MAP were significantly lower in the intervention group (HR: 100.2 ± 8.8 *vs.* 108.5 ± 9.2 bpm; *t* = −6.785, *p* < 0.001; MAP: 83.1 ± 6.7 *vs.* 88.4 ± 7.1 mmHg; *t* = −5.039, *p* < 0.001). Similar trends were observed in the recovery phase (HR: 93.2 ± 7.6 *vs.* 98.1 ± 8.4 bpm, *t* = −4.181, *p* < 0.001; MAP: 77.1 ± 5.8 *vs.* 80.8 ± 6.4 mmHg, *t* = −4.101, *p* < 0.001). Taken together, these findings suggest that Comfort Theory–based nursing not only reduced subjective anxiety but also suppressed physiological manifestations of stress, promoting cardiovascular stability during perioperative care.

### Reduction of postoperative pain intensity

**Figure 4:**
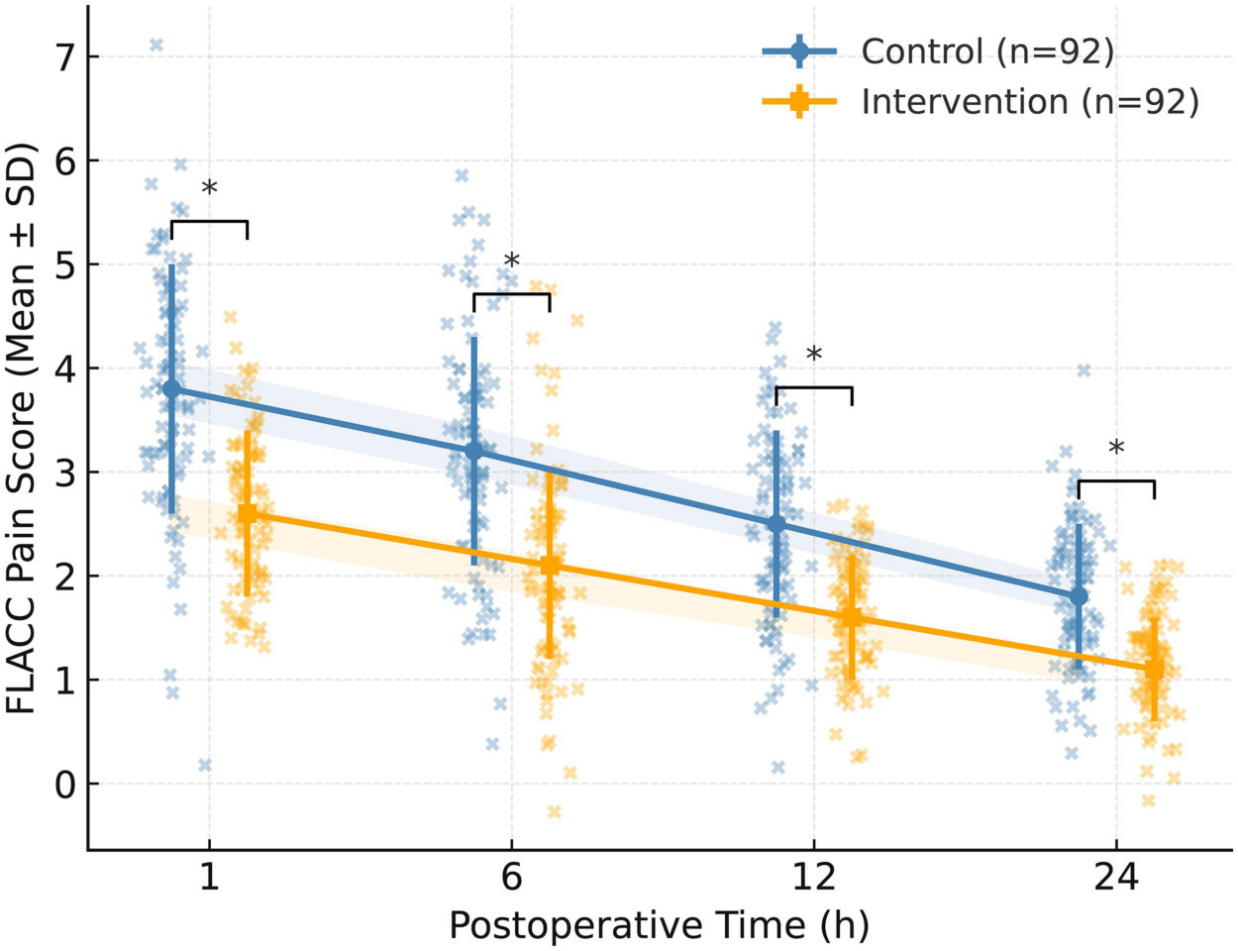
Postoperative pain trajectories measured by the face–legs–activity–Cry–consolability (FLACC) scale over 24 h. Mean FLACC scores were significantly lower in the intervention group across all postoperative time points (1, 6, 12, and 24 h; all *p* < 0.001). Linear mixed-effects modeling confirmed a significant group  ×  time interaction (*F* = 14.589, *p* < 0.001). Pain declined faster in the intervention group. * indicates *p* < 0.05.

Postoperative pain outcomes, measured by the FLACC behavioral scale at 1, 6, 12, and 24 h after surgery, are presented in Table 2 and Figure 4. At each observation point, pain scores were significantly lower in the intervention group (all *p* < 0.001): 1 h: 2.6 ± 0.9 *vs.* 3.8 ± 1.1 (*t* = −8.171, *p* < 0.001, d = 0.93); 6 h: 2.1 ± 0.8 *vs.* 3.2 ± 1.0 (t = −8.186, *p* < 0.001, d = 0.96); 12 h: 1.6 ± 0.7 *vs.* 2.5 ± 0.9 (*t* = −7.432, *p* < 0.001, d = 0.93); 24 h: 1.1 ± 0.6 *vs.* 1.8 ± 0.8 (*t* = −6.172, *p* < 0.001, d = 0.80). Linear mixed-effects modeling confirmed a significant group  ×  time interaction (*F* = 14.589, *p* < 0.001), indicating a faster pain decline trajectory and sustained analgesic benefit in the Comfort Theory group. These findings underscore that the comfort-centered approach alleviated postoperative discomfort by reducing both emotional and physical distress.

**Table 2:** Comparison of postoperative pain (FLACC) and behavioral recovery (PHBQ) between groups.

Outcome	Time point	Intervention group (mean ± SD)	Control group (mean ± SD)	*t*/Z	*p*-value
FLACC pain score	1 h	2.6 ± 0.9	3.8 ± 1.1	−8.171	<0.001
	6 h	2.1 ± 0.8	3.2 ± 1.0	−8.186	<0.001
	12 h	1.6 ± 0.7	2.5 ± 0.9	−7.432	<0.001
	24 h	1.1 ± 0.6	1.8 ± 0.8	−6.172	<0.001
PHBQ score (Post-op Day 3)		7.4 ± 3.1	10.2 ± 3.7	−5.212	<0.001

Postoperative pain intensity was evaluated using the FLACC scale at 1, 6, 12, and 24 h after surgery, while behavioral adaptation was assessed on postoperative day 3 using the Post-Hospitalization Behavior Questionnaire (PHBQ). Children receiving Comfort Theory–guided nursing care demonstrated significantly lower pain scores across all time points and better postoperative behavioral recovery compared with the control group (all *p* < 0.001). FLACC, Face–Legs–Activity–Cry–Consolability pain scale; PHBQ, Post-Hospitalization Behavior Questionnaire.

### Improvement in behavioral recovery and emotional adaptation

Behavioral adaptation following hospitalization was evaluated using the PHBQ scale on postoperative day 3. Children in the intervention group displayed significantly fewer negative behavioral changes, including separation anxiety, sleep disturbance, and regression, than those receiving routine care. The mean PHBQ score was 7.4 ± 3.1 in the intervention group vs. 10.2 ± 3.7 in controls (*Z* = −5.212, *p* < 0.001, *r* = 0.380), representing a 27.5% relative reduction in maladaptive behaviors. This improvement reflects better emotional adjustment and psychological recovery, consistent with the theoretical framework of enhanced comfort and security.

### Enhanced recovery efficiency and parental satisfaction

Functional recovery indicators revealed marked benefits in the intervention group (Table 3). Children receiving Comfort Theory–guided care regained full consciousness earlier (19.6 ± 3.8 *vs.* 22.4 ± 4.1 min; *t* = −4.449, *p* < 0.001) and resumed oral intake more rapidly (4.9 ± 1.0 *vs.* 5.8 ± 1.2 h; *t* = −5.192, *p* < 0.001). Their mean length of hospital stay was significantly shorter (3.4 ± 0.7 *vs.* 3.9 ± 0.8 days; *t* = −4.727, *p* < 0.001). Parental satisfaction scores also favored the intervention group (4.8 ± 0.4 *vs.* 4.3 ± 0.6; *t* = 6.283, *p* < 0.001). Qualitative feedback collected from post-discharge interviews (not statistically analyzed) echoed these results, with parents frequently emphasizing the nurses' empathetic communication, structured comfort measures, and improved child cooperation as key contributors to their satisfaction. These findings highlight that psychological comfort interventions can translate into tangible efficiency gains and superior family-centered care experiences.

**Table 3:** Comparison of recovery efficiency and parental satisfaction.

Parameter	Intervention group (*n* = 92)	Control group (*n* = 92)	*t*	*p*-value
Time to awakening (min)	19.6 ± 3.8	22.4 ± 4.1	−4.449	<0.001
Time to first oral intake (h)	4.9 ± 1.0	5.8 ± 1.2	−5.192	<0.001
Length of hospital stay (days)	3.4 ± 0.7	3.9 ± 0.8	−4.727	<0.001
Parental satisfaction (1–5 likert)	4.8 ± 0.4	4.3 ± 0.6	6.283	<0.001

This table presents postoperative recovery indices and parental satisfaction outcomes. The Comfort Theory–guided group exhibited significantly shorter awakening and oral intake times, reduced hospital stay, and higher parental satisfaction scores than the control group (all *p* < 0.001), indicating more efficient recovery and enhanced family-centered care experience. h, hours; min, minutes; SD, standard deviation.

### Safety profile and adverse events

No severe adverse events occurred during the study period. The incidence of minor postoperative complications was low and comparable between groups: nausea or vomiting (7.6% *vs.* 9.8%; *p* > 0.05), emergence agitation (5.4% *vs.* 8.7%; *p* > 0.05), and superficial wound infection (0% *vs.* 1.1%; Fisher's exact test, *p* > 0.05). All cases were self-limiting or resolved with standard symptomatic management. These data confirm that implementing Comfort Theory–based care did not increase perioperative risks and was safe for pediatric patients.

## Discussion

The present study suggests that Comfort Theory–guided nursing care may reduce perioperative anxiety, mitigate physiological stress responses, alleviate postoperative pain, and support behavioral and functional recovery among pediatric patients undergoing strabismus surgery. By systematically addressing the physical, psychospiritual, sociocultural, and environmental dimensions of comfort, this approach yielded significant clinical and psychological benefits beyond those achieved through routine nursing (19). The results support the core proposition of Katharine Kolcaba's Comfort Theory that intentional, multidimensional comfort interventions can produce measurable improvements in patient well-being and recovery outcomes (14). To our knowledge, this is among the first controlled clinical investigations to empirically validate the theoretical model of comfort in a pediatric ophthalmic surgical setting, thereby extending its application from conceptual framework to evidence-based practice.

These findings suggest that structured, theory-driven comfort nursing may be considered as a supportive perioperative strategy for children undergoing ophthalmic surgery, particularly those at high risk of preoperative anxiety. However, the present intervention was implemented as a multimodal bundle incorporating psychological preparation, environmental modification, parental involvement, and continuous emotional support. As such, the study design does not allow for isolation of individual component effects. The observed benefits likely reflect the combined and potentially synergistic impact of these elements, consistent with the holistic nature of Comfort Theory. The implementation of this integrated model was associated with significantly lower m-YPAS anxiety scores before anesthesia and during early postoperative recovery. Concurrent reductions in heart rate and mean arterial pressure suggest that the intervention not only alleviated perceived anxiety but also modulated physiological stress responses (17, 20). These findings indicate that psychological comfort may translate into measurable physiological stabilization in pediatric perioperative care. At the same time, the magnitude of the observed effect sizes, particularly for m-YPAS, warrants careful consideration. Compared with prior studies of non-pharmacological interventions, the effect sizes in this study are relatively large. This may reflect the influence of behavioral measurement characteristics, sustained caregiver engagement, and contextual factors inherent to comfort-based interventions. Such factors may also contribute to an overestimation of intervention effects and should be considered when interpreting the findings. In addition, consistent reductions in FLACC pain scores over 24 h postoperatively suggest that comfort-oriented nursing may indirectly enhance analgesic efficacy, potentially by reducing anxiety-related pain amplification. Collectively, these results support the concept that comfort is a dynamic state influencing both emotional experience and physiological resilience, thereby reinforcing Kolcaba's theoretical postulate that comfort promotes healing and adaptive responses (21).

Beyond validating the practical benefits of Comfort Theory, this study contributes to the broader advancement of pediatric perioperative nursing science in three major ways. First, it bridges the conceptual gap between holistic nursing theory and quantitative outcome evaluation. Previous comfort-care research was often descriptive or limited to subjective satisfaction metrics; by integrating validated behavioral (m-YPAS, PHBQ) and physiological indicators, this study provides quantitative supportive evidence for the potential benefits of Comfort Theory–guided nursing (22, 23). Second, it enriches the methodological repertoire for pediatric ophthalmology by introducing a reproducible, theory-based intervention that can be adapted across diverse clinical contexts. The structured comfort assessment, environmental adjustment, parental participation, and emotional-coaching strategies form a standardized yet flexible framework applicable to other anxiety-provoking pediatric procedures such as tonsillectomy or MRI sedation. Third, the observed improvements in recovery-related outcomes, including awakening time, time to oral intake, and length of hospital stay, should be interpreted with a clear distinction between statistical significance and clinical relevance. Although these differences reached statistical significance, the absolute magnitude of change was modest (e.g., approximately 3 min for awakening time and 0.5 days for hospital stay). From a clinical perspective, the relevance of these differences may be context-dependent. In pediatric perioperative care, even small reductions in early recovery time may help decrease agitation, crying episodes, and the need for additional nursing interventions during the vulnerable emergence period. Earlier oral intake may also improve hydration status and reduce parental concern, thereby facilitating smoother postoperative management. At the institutional level, incremental reductions in recovery time and hospital stay may cumulatively improve bed turnover efficiency and resource utilization in high-volume settings. Nevertheless, these findings should not be overinterpreted. The distinction between statistical significance and clinically meaningful benefit should be carefully considered when translating these results into practice.

Despite its strengths, including randomized allocation, blinded outcome assessment, and comprehensive multidimensional evaluation, several limitations should be acknowledged. First, this was a single-center study conducted in a tertiary hospital with a relatively homogeneous patient population, which limits external validity. Differences in healthcare organization, perioperative workflows, and family involvement across institutions and countries may influence the feasibility and effectiveness of comfort-based interventions. In particular, the longer hospital stay observed in this study, compared with day-case surgery commonly reported internationally, reflects local healthcare policies and perioperative practices in China. Therefore, caution is required when extrapolating these findings to other healthcare systems, and multicenter studies are needed to confirm generalizability. Second, although major perioperative variables were standardized, several psychosocial factors known to strongly influence pediatric perioperative anxiety, including parental anxiety, prior hospitalization experience, and socioeconomic background, were not quantitatively assessed or adjusted for. These factors may have influenced the observed outcomes and introduce the possibility of residual confounding. Third, the single-blind design, in which outcome assessors were blinded but participants, parents, and nursing staff were not, introduces a risk of performance and expectation bias. In addition, because primary outcomes such as m-YPAS and PHBQ are based on behavioral observation, observer-dependent outcome inflation cannot be excluded. These factors may have contributed to an overestimation of the intervention effect, particularly given the relatively large effect sizes observed. Fourth, the intervention was delivered as a multimodal bundle incorporating psychological preparation, environmental modification, parental involvement, and emotional support. As such, the relative contribution of individual components could not be determined, which limits interpretability and reproducibility. Future studies using factorial or mediation designs are needed to clarify the contribution of specific components. Fifth, the study evaluated only short-term outcomes up to postoperative day 3. Longer-term outcomes, including sustained emotional adaptation and adherence to postoperative care, were not assessed and warrant further investigation. Finally, although validated instruments were used, behavioral scales such as m-YPAS and PHBQ rely partly on observer interpretation and may not fully capture children's internal experiences. In addition, minimally clinically important differences for these measures have not been well established in pediatric perioperative populations, and therefore the clinical significance of the observed changes should be interpreted with caution. Future studies incorporating objective biomarkers of stress, together with standardized fidelity monitoring, may further strengthen the evidence base.

Importantly, comfort-based nursing should be regarded as an adjunctive perioperative strategy rather than a replacement for standard anesthetic management or pharmacological anxiolysis when clinically indicated. The practical implications of this study extend across pediatric surgical, anesthetic, and nursing domains. In clinical practice, Comfort Theory–based nursing offers a standardized yet patient-centered framework that can be easily integrated into existing perioperative protocols without additional equipment or cost. By emphasizing environmental modification, communication, and family engagement, this model aligns with modern family-centered care principles and enhances interdisciplinary collaboration between nurses, anesthesiologists, and surgeons. From an educational standpoint, incorporating Comfort Theory into nursing curricula can cultivate reflective practice and empathy, transforming comfort provision from intuitive behavior into a structured competency. Simulation-based training could reinforce communication and emotional-support techniques, thereby improving pediatric care quality. Administratively, the demonstrated reductions in hospital stay and improved parental satisfaction suggest that comfort-guided nursing contributes to institutional quality metrics and patient-experience scores, which are increasingly tied to healthcare reimbursement and accreditation standards. Moreover, the universality of this theory makes it adaptable beyond ophthalmology, including pediatric oncology, orthopedics, and intensive care, where anxiety and procedural distress are common. The conceptual clarity of relief, ease, and transcendence allows outcome-based customization, enabling institutions to design comfort interventions tailored to disease-specific stressors while maintaining theoretical consistency. In the broader scope of nursing science, this study supports a paradigm shift from task-oriented to experience-oriented care, demonstrating that intentional comfort cultivation is both scientifically valid and clinically impactful.

Building on these findings, future research should aim to expand both scope and depth of comfort-based nursing evaluation. Large-scale multicenter randomized trials across different surgical specialties would establish generalizability and allow subgroup analyses based on age, temperament, and parental anxiety levels. Longitudinal designs following patients over several months could explore whether reduced perioperative anxiety translates into sustained behavioral resilience and better visual outcomes after strabismus correction. Intervention refinement should focus on identifying the most influential components of comfort care, such as parental presence, play therapy, and environmental adjustments, using factorial or mediation analyses to guide efficient resource allocation. Furthermore, the development of digital comfort-assessment tools and standardized documentation systems could facilitate routine clinical integration and real-time quality monitoring. At a theoretical level, future studies could explore cross-theory synthesis between Comfort Theory and constructs such as self-efficacy or attachment theory to generate comprehensive models of pediatric coping. Finally, given the increasing emphasis on patient-reported outcomes and compassionate care within global healthcare frameworks, the adoption of Comfort Theory–guided nursing could serve as a model for value-based pediatric care, bridging humanistic values with measurable clinical performance. By systematically validating comfort as a therapeutic outcome, nursing science can continue to lead interdisciplinary efforts to humanize medicine and improve pediatric surgical recovery worldwide.

## Conclusion

This study suggests that Comfort Theory–guided nursing care may reduce perioperative anxiety, stabilize physiological stress responses, alleviate postoperative pain, and facilitate early recovery in children undergoing strabismus surgery. By addressing comfort across physical, psychospiritual, sociocultural, and environmental domains, this approach may offer a practical and holistic framework for optimizing pediatric perioperative care. However, given the single-center design, the absence of full blinding, and the multimodal nature of the intervention, these findings should be interpreted with caution, and further studies are needed to confirm their generalizability and to clarify the contribution of individual components.

## Data Availability

The raw data supporting the conclusions of this article will be made available by the authors, without undue reservation.
